# Supplementary material for: Assessing alternative indicators for Covid-19 policy evaluation, with a counterfactual for Sweden
Source: PLoS One. 2022 Mar 16;17(3):e0264769. doi: 10.1371/journal.pone.0264769 (PMC8926176; doi:10.1371/journal.pone.0264769)
Supplement: Attachment — Submitted filename: Response to Reviewers.pdf [file pone.0264769.s001.pdf]

Stockholm, 19/10/2021

Dear Editor,

Thank you very much for allowing us to revise our manuscript "Assessing Alternative Indicators for Covid-19 Policy Evaluation, with a Counterfactual for Sweden" for publication on PLOS ONE. Please see below the comments of each reviewer followed in red with how we have taken them into account in this revision. We remain of course available for any further clarification.

With our best regards,

Giancarlo Spagnolo (for all the authors)

Reply to reviewer #1

1. Abstract and introduction section has written clearly.

2. Section '2.2 Heterogeneity over time', please report the sources of the following statement, ... "Prior to March 12, 2020, the Swedish strategy was to test all people who had been in areas considered at risk, like China and Italy, but due to shortages in testing equipment, this strategy was rapidly changed, and testing was only targeted towards people with heavy symptoms and in need of hospitalization and medical care staff." "Sweden then reversed this policy announcing on March 31, 2020, a plan to expand testing capacity to all critical services: the aim was to carry out 100,000 tests per week."
Corrected.

3. Section 'Methodology'. The author(s) has clearly explained the synthetic control method.

4. Section '4 Data' data analysis has been explained clearly.

5. Section '5 Results' has discussed clearly and supported with previous literature.

6. Please include the practical and managerial implication section before conclusion.
This is done in the new Section 6.

7. Please include the limitation and future study, and highlight the impacts/values of this study in the society.
This is done in the new Section 6.

Reply to reviewer #2

1. The effect of lockdown against the spread of the COVID-19 pandemic depends on many factors, including lifestyle of people in different countries, the density of population, sizes of cities, time of taking lockdown measure, and so on. All this kinds of problems were not taken into account or discussed in this paper. Because of the above problems, it is very doubtful whether the conclusion of the paper is general.
These problems are now discussed in the new Section 6.

2. Eight indicators were employed in this studies. However, the authors did not discuss the relationships between these indicators. In data analysis, adding an indicator will bring information and noise at the same time. When using multiple indicators for quantitative analysis, you must pay attention to the orthogonality and dimensional consistency between indicators. In addition, the effectiveness of indicators and the redundancy of indicator system should be discussed.
If we understand correctly, the reviewer is concerned with the precise definition and units of measures of the different indicators. These are now provided in Sections 4.1 and 4.2. As for the orthogonality/correlation between the different indicators, please see the discussion in Section 5.1.

3. Some places are unclear. For example, there are no substantial differences between the titles and notes of Figures 4, 5 and 6.
Thanks for noting this. We corrected the titles of Figures 4 and 5 (Figure 6 is clearly different).

4. The paper is poorly written by an author whose first language is clearly not English. In fact, there are too many problems in the grammar, usage, and overall readability of the manuscript. The manuscript should be revised to fix the grammatical errors and improve the overall readability of the text before it is accepted for possible publication. I suggest the author(s) have a fluent, preferably native, English-language speaker thoroughly copyedit this manuscript for language usage, spelling, and grammar.
The revised version of the manuscript was proof-read by a native English speaker who is familiar with the language of economists/demographists.

Reply to reviewer #3

1.The core of the paper is not reflected in its title which suggests – as also mentioned in the first sentence of the introduction that – that it has a much broader focus, namely to analyze "what indicators should be used to monitor epidemics and evaluate policies, such as lockdowns or other nonpharmaceutical interventions." However, the latter question is not addressed in the paper. For sure, in section 4 the authors introduce the "adjusted COVID-19 deaths" indicator. Moreover, somewhat related, but already very much focused on Sweden, the paper discusses the relationship between the number of reported infections and testing intensity which leads to the identification of the "positive rate" as another indicator to measure the impact of NPIs and lockdowns. However, the indicator itself has been widely discussed in the literature before (see e.g. Hasell et al. (2020). A cross-country database of COVID-19 testing, Scientific data, 7(1), 1-7). More importantly, I do not find supporting evidence for the claim that "looking at several indicators", including adjusted deaths and the positive rate, is needed to "derive robust conclusions" (on what? on the COVID-19 effects of lockdowns?), also because the analysis is just a case study of Sweden. Thus, I tend to disagree with conclusion that the "study highlights the importance of looking at multiple indicators when evaluating policies or comparing countries" (page 17). Accordingly, I recommend that the authors change the title and the introduction/conclusion accordingly, focus on the Swedish case and refrain from making general statements suggesting that the paper is about the pros and cons of several indicators measuring the state of a pandemic.

The introduction and conclusion have been changed to better reflect the focus of the paper. However, considering multiple indicators our study shows that two previous studies on Sweden focussing on a single, problematic indicator derived unreliable conclusions on the delay with which a lockdown would have begun to have an effect (four time longer that what all other indicators consistently suggest). We believe this result indeed "highlights the importance of looking at multiple indicators when evaluating policies or comparing countries" to reach more reliable/robust conclusions.
The need of looking at several indicators is justified by the problems raised in Sections 2.1 and 2.2 where we discuss the differences in testing intensity and deaths recording across countries, in Section 4.1 where we discuss other problems related to infections, and in Section 4.2 where we discuss the issues with excess mortality. It is also supported by our results in Section 5.2 where we discuss the problems related to the lag between deaths and infections. The problems discussed in the just mentioned question are not only related to Sweden but at least most of the European countries.
We have discussed the issue of the external validity of our results at the end of the new section 6.

2.With the core of the paper being the Swedish case, the paper basically aims at reconsidering the results obtained by Born et al. (2020) and Cho (2020) with some new indicators (the "adjusted COVID-19 deaths" and the "positive rate") as well as cumulative versus daily data. Indeed, most of the paper can be seen as a kind of robustness check and extension of Born et al. (2020) and Cho (2020). It does a good job in doing so and in qualitative terms confirms the results of previous studies. Thus, I recommend more restraint when discussing the novelty of the results (this holds even more when considering the 2021 version of Born et al. (2020) published in PLOS One https://journals.plos.org/plosone/article?id=10.1371/journal.pone.0249732 given the extensions, also in terms of the post-treatment period considered, compared to the 2020 version). Indeed, for death indicators the results seem to be broadly the same as in Born et al. (2020) and Cho (2020). Things are different for infections. However, even here the paper's main new insight – that a Swedish lockdown would have worked faster than previous research suggests – is mainly driven by the positive rate and when making use of daily instead of cumulative cases.

We have deemphasized the novelty of our results where necessary.

3. The paper develops the "adjusted COVID-19 deaths" indicator. As shown in section 4.3 it adds to the number of reported COVID-19 deaths excess mortality, if excess mortality is positive. The paper argues that such an adjustment is needed as "the number of reported COVID-19 deaths is likely to represent a downward biased estimate of total COVID-19 deaths" (p. 9). However, I do not find arguments why this should be the case. Indeed, on pages 2 and 3 arguments are presented suggesting that reported COVID-19 cases might be overreported (with Sweden itself being a candidate as it "has been rather generous in ascribing deaths to COVID-19" (p. 3). Moreover, excess mortality is unlikely to be affected by COVID-19 deaths only (as stated in the form of an assumption on page 9) but also by nonpharmaceutical interventions themselves which can raise but also lower mortality rates independently from reported COVID-19 deaths. Moreover, these effects are likely to vary across countries, for example due to different demographic characteristics. Thus, I have doubts whether the claim made on the bottom of page 8 is correct, namely that "excess mortality is … robust to structural differences across countries." Accordingly, I would welcome a more balanced discussion about the advantages and disadvantages of the adjusted compared to unadjusted COVID-19 deaths.

We have provided a reference to support our argument "the number of reported COVID-19 deaths is likely to represent a downward biased estimate of total COVID-19 deaths".
Overreporting cases of COVID-19 deaths (such as for the case of Sweden) are rare examples of outliers which are being corrected in the COVID-19 deaths statistics by the responsible agencies in the next days.
We have introduced a more balanced discussion of the advantages and disadvantages of excess mortality which affects adjusted COVID-19 deaths in section 4.2.
We assumed that COVID-19 is the only IMPORTANT (*and not the only*) cause of HIGHER mortality in 2019. On page 8, third paragraph, we list other things that can affect mortality.

Minor points:

- The direct quotes on page 3 are pretty lengthy and could be summarized by the authors in a shorter way.
Done.

- Figures are presented without reference to data sources. This should be corrected.
Corrected.

- Figure 1: Typo: inhabitants.
Corrected.

- Page 5, second para, first sentence: Typo: "Sweden finally managed to step up …
Corrected.

- Page 5: it is not clear to me why the risk of overfitting may increase with the size of the donor pool, especially when T0 is small (I assume T_0 refers to the length of the pre-intervention period).
As explained by Abadie 2021 (p. 401), "The risk of over-fitting may also increase with the size of the donor pool, especially when T_0 is small. For any fixed T_0, a larger J makes it easier to fit pretreatment outcomes even when there are substantial discrepancies in factor loadings between the treated unit and the synthetic control. Consistent with this argument, the bias bound for $\hat{\tau}_{1t}$ derived in Abadie, Diamond and Hainmuller (2010) depends positively on J."

- Page 10, footnote 11: Please clarify whether the footnote refers to the baseline, the robustness checks or both.
Corrected.

- Page 10, footnote 13: The message given is unclear without having read Cho (2020).
Corrected.

- Page 11, Section 5.2, second para: Is "overreporting" the only reason for Sweden showing a slightly smaller number of COVID-19 deaths than its synthetic counterpart?
As argued in Section 5.2, it is one possible explanation: "This difference could be associated with anomalies

in recorded COVID-19 deaths in early April when, on April 4 (day 18), Sweden reported a negative and relatively large number of deaths, most likely with the intent to correct over-reporting in previous days".

- Page 12, second para, last sentence: Is the variance argument not implying that COVID-19 deaths are in general not a useful measure for assessing the speed of the impact of NPIs and lockdowns?
Yes, this is exactly the conclusion we draw. We believe that COVID-19 deaths are a good measure to evaluate the total effect of the lockdown but not the speed of its impact.

- Page 12, last para: the sentence "While the curves … as in Born et al. (2020) and Cho (2020)" does not seem to have a main clause.
Corrected.

- Page 14, last para: are the reported differences between results obtained in this paper compared to Born et al. (2020) and Cho (2020) significant?
Confidence intervals and tests of significance are not available for the synthetic control method and the standard methods for calculating the statistical significance of the post-treatment outcome cannot be used in this case. Thus, robustness checks (such as those presented in Section 5.3) are particularly important.

Changes to the reference list

Added references:

European Centre for Disease Prevention and Control (ECDC) (2021). Data on testing for COVID-19 by week and country. https://www.ecdc.europa.eu/en/publications-data/covid-19-testing.

Edwards, C. (2020). How many people is Sweden testing for the Coronavirus and how does that compare globally? *The Local*. https://www.thelocal.se/20200506/how-many-people-is-sweden-testing-for-the-coronavirus-and-how-does-that-compare-globally/.

Kluge H. H. (2020). Statement – Digital health is about empowering people. *World Health Organization*. https://www.euro.who.int/en/media-centre/sections/statements/2020/statement-digital-health-is-about-empowering-people.

Government Offices of Sweden (2020). https://www.government.se/49eca8/globalassets/government/dokument/utrikesdepartementet/diplomatportalen/update-testing-covid-19.pdf.

Peracchi, F., and D. Terlizzese (2020). Estimating the prevalence of the COVID-19 infection, with an application to Italy. *Covid Economics 43 (21 August 2020),* 19-41. https://cepr.org/content/covid-economics-vetted-and-real-time-papers-0#block-block-10.

Socialstyrelsen (2020). Description of data sources for deaths due to COVID-19. https://www.socialstyrelsen.se/globalassets/1-globalt/covid-19-statistik/engelska-sidan/faktablad-datakallor-eng.pdf.

The Economist (2021). Tracking COVID-19 deaths across countries. https://www.economist.com/graphic-detail/coronavirus-excess-deaths-tracker.

FT Visual and Data Journalism team (2021). Coronavirus tracker: the latest figures as countries fight the COVID-19 resurgence. *Financial Times*. https://www.ft.com/content/a2901ce8-5eb7-4633-b89c-cbdf5b386938.

Manski, C. F., and F. Molinari (2021). Estimating the covid-19 infection rate: Anatomy of an inference problem. *Journal of Econometrics 220(1)*, 181–192. https://www.sciencedirect.com/science/article/pii/S0304407620301676.

Dropped references:

Roser, M., H. Ritchie, E. Ortiz-Ospina, and J. Hasell (2020). Coronavirus pandemic (COVID-19). *Our World in Data*. https://ourworldindata.org/coronavirus.

Aron, J. and J. Muellbauer (2020). A pandemic primer on excess mortality statistics and their comparability across countries. *Our World in Data*.https://ourworldindata.org/excess-mortality-covid.

Modified references:

Abadie, A. (2021). Using synthetic controls: Feasibility, data requirements, and methodological aspects. *Journal of Economic Literature 59(2)*, 391-425.

Faes, C., S. Abrams, D. Van Beckhoven, G. Meyfroidt, E. Vlieghe, and N. Hens (2020). Time between symptom onset, hospitalisation and recovery or death: statistical analysis of Belgian COVID-19 patients. *International Journal of Environmental Research and Public Health 17*.

https://doi.org/10.3390/ijerph17207560.

Fowler, J. H., J. S. Hill, and N. Obradovich (2021). Stay-at-home orders associate with subsequent decreases in COVID-19 cases and fatalities in the United States. *PloS ONE 16(6)*. https://doi.org/10.1371/journal.pone.0248849*v*.

Rahi, A. and B. Heydari (2021). The immediate effect of COVID-19 policies on social-distancing behavior in the United States. Public Health Reports 136(2), 245-252. https://journals.sagepub.com/doi/full/10.1177/0033354920976575

Rees, E. M., E. S. Nightingale, Y. Jafari, N. R. Waterlow, S. Clifford, C. A. B. Pearson, T. Jombart, S. R. Procter, and G. M. Knight (2020). Covid-19 length of hospital stay: a systematic review and data synthesis. *BMC MED 18, 270* . https://doi.org/10.1186/s12916-020-01726-3

Ritchie, H. and Mathieu, E and Rodé -Guirao, C. Appel, C. E. Giattino, Ortiz-Ospina, J. Hasell, B. Macdonald, B. BD, and M.Roser (2020). Coronavirus pandemic (COVID-19). *Our World in Data*. https://ourworldindata.org/coronavirus.
