# Supplementary material for: Assessing alternative indicators for Covid-19 policy evaluation, with a counterfactual for Sweden
Source: PLoS One. 2022 Mar 16;17(3):e0264769. doi: 10.1371/journal.pone.0264769 (PMC8926176; doi:10.1371/journal.pone.0264769)
Supplement: Attachment — Submitted filename: Response to Reviewers2 .pdf [file pone.0264769.s002.pdf]

Review Comments to the Author

Reviewer #1: The author has improved the paper accordingly but there is still lack of information about the practical contribution and impact of the finding in the society. The paper needs to explain some potential limitation before final accepting the paper for publishing.

We have added the practical implications and limitations of our study in the last two paragraphs of the conclusions.

Reviewer #3: Dear authors,
thank you for the revised version of the paper which takes on board most of my concerns I raised in the first review. Only a few minor points are left:
Page 1, third para, line 8. Typo: "… the number of recorded infections to the number of tests …"
Corrected
Page 1, fourth para, line 4. Suggestion for using a less strong language, i.e. something like: "This finding contrasts with the longer lags (three to five weeks) in the effect of lockdowns estimated in Born et al. (2020) and Cho (2020) …
Corrected
Page 1, fourth para, last line: Which are the other studies you have in mind showing results which are more consistent with you study?
Corrected
Page 4, Heterogeneity over time: This subsection, as it stands, only deals with testing policy (Is this the only heterogeneity over time?). If this remains the case the subtitles should reflect that "2.1 Heterogeneity across countries" deals with various issues (ascribing COVID deaths and testing), while 2.2. only deals with testing.
Corrected
Page 4, second para: who are the "people who had been in areas considered at risk"? Could you be a bit more explicit?
Corrected
Page 4, third para: Wording suggestion "… those worried about their infection states but unable to be tested before, hence exhibited a higher probability …."
Corrected
Page 7, second para: Wording suggestion: "… argue that excess mortality is more comparable across countries because it is less sensitive …"
Corrected
Page 10, last para, second line: Suggestion for toning down language: "… our estimates of the effects of a lockdown are often somewhat higher than in previous works." (there is one case, where your effect is less strong than in Cho (2020). Moreover, given that tests of significance cannot be run, I think that introducing "somewhat" is a way of expressing that the difference between numbers like 34 and 40% (cumulative COVID deaths) is not that large.
Corrected

Additional comment:
Regarding the answers 'NO' to the question whether we made our data available:
All data we use are publicly available. The sources of the data are described in Section 4 of our paper and are the following:
Our World in Data - Covid deaths and number of infections
European Centre for Disease Prevention and Control ECDC - Covid tests
Financial Times - excess mortality
